# PD-L1 Inhibitor Regulates the miR-33a-5p/PTEN Signaling Pathway and Can Be Targeted to Sensitize Glioblastomas to Radiation

**DOI:** 10.3389/fonc.2020.00821

**Published:** 2020-05-27

**Authors:** Wenzheng Xia, Jin Zhu, Yinda Tang, Xueyi Wang, Xiangyu Wei, Xuan Zheng, Meng Hou, Shiting Li

**Affiliations:** ^1^Department of Neurosurgery, Xinhua Hospital Affiliated to Shanghai Jiaotong University School of Medicine, Shanghai, China; ^2^Department of Radiation Oncology, First Affiliated Hospital, Wenzhou Medical University, Wenzhou, China

**Keywords:** glioblastoma, programmed death ligand 1 (PD-L1) blockade, radio-sensitization, miR-33a-5p/PTEN signaling pathway, DNA damage response

## Abstract

Glioblastoma (GBM) is the most common and lethal brain tumor in adults. Ionizing radiation (IR) is a standard treatment for GBM patients and results in DNA damage. However, the clinical efficacy of IR is limited due to therapeutic resistance. The programmed death ligand 1 (PD-L1) blockade has a shown the potential to increase the efficacy of radiotherapy by inhibiting DNA damage and repair responses. The miR-33a-5p is an essential microRNA that promotes GBM growth and self-renewal. In this study, we investigated whether a PD-L1 inhibitor (a small molecule inhibitor) exerted radio-sensitive effects to impart an anti-tumor function in GBM cells by modulating miR-33a-5p. U87 MG cells and U251 cells were pretreated with PD-L1 inhibitor. The PD-L1 inhibitor-induced radio-sensitivity in these cells was assessed by assaying cellular apoptosis, clonogenic survival assays, and migration. TargetScan and luciferase assay showed that miR-33a-5p targeted the phosphatase and tensin homolog (PTEN) 3′ untranslated region. The expression level of PTEN was measured by western blotting, and was also silenced using small interfering RNAs. The levels of DNA damage following radiation was measured by the presence of γ-H_2_AX foci, cell cycle, and the mRNA of the DNA damage-related genes, BRCA1, NBS1, RAD50, and MRE11. Our results demonstrated that the PD-L1 inhibitor significantly decreased the expression of the target gene, miR-33a-5p. In addition, pretreatment of U87 MG and U251 cells with the PD-L1 inhibitor increased radio-sensitivity, as indicated by increased apoptosis, while decreased survival and migration of GBM cells. Mir-33a-5p overexpression or silencing PTEN in U87 MG and U251 cells significantly attenuated PD-L1 radiosensitive effect. Additionally, PD-L1 inhibitor treatment suppressed the expression of the DNA damage response-related genes, BRCA1, NBS1, RAD50, and MRE11. Our results demonstrated a novel role for the PD-L1 inhibitor in inducing radio- sensitivity in GBM cells, where inhibiting miR-33a-5p, leading to PTEN activated, and inducing DNA damage was crucial for antitumor immunotherapies to treat GBM.

## Introduction

Glioblastomas (GBM) are one of the most treatment-resistant tumors, often recurring after chemotherapy and radiation treatment (1). Amount of effort has been taken to identify therapeutics that radio-sensitize GBMs because most patients will receive radiation treatment (2). However, it's difficult to identify such radiosensitive chemotherapeutic agents because GBMs exhibit redundant pro-growth and pro-survival pathways, leading to chemotherapy resistance (3). To overcome such resistance, it's needed to devise therapeutic strategies targeting such redundant treatment-resistant pathways to increase the radiosensitivity of GBMs.

Programmed death ligand 1 (PD-L1)regulates the immune system by binding the programmed cell death protein 1 (PD-1) receptor as an immune checkpoint protein (4, 5). By combining with PD-1 on immune cells, PD-L1 helps tumor cells escape from the immune system and survive (6). Therefore, abrogation of the PD-1 and PD-L1 interaction has acted as an effective therapeutic strategy to enhance antitumor immunity across multiple malignancies. The immune checkpoint blockade has shown a therapeutic effect in immunosuppressive GBMs (7). With respect to radiosensitization in colorectal carcinoma and breast cancer cell lines, knockdown of PD-L1 sensitizes cells to radiotherapy (8). Although the impact of the PD-L1 blockade on radiosensitization has been suggested in GBM, its role has yet to be fully elucidated.

MicroRNAs (miRNAs) are a series of small, noncoding RNA molecules, typically about 18–22 nucleotides in length in the mature form (9). miRNAs negatively regulate gene expression at the post-transcriptional level by inhibiting mRNA translation and/or promoting mRNA degradation (10). In recent years, abundant miRNAs have been found to be deregulated in many types of cancer: some function as tumor suppressors and others as tumor promoters (11). The miR-33a-5p is located on chromosome 22, and its high expression is related with the poor prognosis of GBM patients (12). miR-33a-5p influences the radiation resistance-associated pathway protein, STAT3, leading to radiation resistance in GBM cells. Importantly, miR-33a-5p is also an essential component of the PTEN regulatory network (13).

PTEN is a critical inhibitor of cell proliferation, viability, and migration in GBMs (14, 15). Following the treatment of cells with PD-1 inhibitors (e.g., pembrolizumab), genomic and transcriptomic analyses revealed a significant accumulation of PTEN mutations, leading to an immunotherapeutic response in GBMs (16). Thus, in the present study, we sought to reveal whether the PD-L1 inhibitor could influence the radiosensitivity of GBM cells by modulating the miR-33a-5p/PTEN pathway.

Genetic alterations involved in GBM progression or recurrence have close relationships with DNA damage response (DDR) (17). DDR contributes to malignancy by regulating diverse cellular functions, including cell metabolism, proliferation and programmed cell death (18). Importantly, the DDR induces chemo- or radio-resistance in GBM (19). Thus, targeting the DDR could promote the growth-suppressive effects of radiation (20). Immune checkpoint blockade using a PD-L1 antagonist targeted the DDR and induced radiosensitization in tumor cells (8). Thus, direct pharmacological targeting of PD-L1 is an attractive approach for sensitizing GBMs to radiation.

Here, we suggested that immune checkpoint blockade using the PD-L1 inhibitor was a potent therapy for GBM radio-sensitization. Furthermore, we showed that the PD-L1 inhibitor induced radiosensitization by modulating the miR-33a-5p/PTEN pathway. Thus, we propose that the immune checkpoint blockade is a promising treatment strategy for GBM radiosensitization.

## Materials and Methods

### Cell Culture and Treatment

The U87 MG human glioblastoma cell line and U251 cells were obtained from the American Type Culture Collection (ATCC). They were cultured in Dulbecco's modified Eagle's medium (DMEM) supplemented with 10% fetal bovine serum and 1% antibiotic-antimycotic solution at 37°C with 5% CO_2_. Experiments were performed with cells grown to 70% confluency.

For the PD-L1 blockade, cells were fed media containing 4 mg/mL PD-L1 inhibitor (Abcam, ab230369) and incubated at 37°C, as previously described (21).

### Ionizing Radiation

Cells were placed in a cesium-137 source irradiator. Cells were irradiated using a single dose of 10 Gy, as previously reported (22).

### Western Blot Analysis

Cells were harvested using RIPA buffer (Sigma-Aldrich). Proteins were separated on 10% SDS-PAGE gels and transferred to polyvinylidene difluoride membranes. The membranes were incubated overnight with the following primary antibodies: anti-PD-L1 (ab205921,1:500), anti-PTEN (ab32199, 1:750), anti-gamma H_2_AX (ab2893, 1:500), and anti-β-actin (ab8227, 1:1000). Following incubation, membranes were washed, incubated for 1 h with appropriate secondary antibodies conjugated to horseradish peroxidase, and developed using chemiluminescent substrates. The stained protein bands were visualized using a Bio-Rad ChemiDoc XRS instrument, and quantified and analyzed using the Quantity One software.

### Flow Cytometric Analysis of Apoptosis

The extent of apoptotic cell death was assayed using the Annexin V-FITC Apoptosis Detection Kit, according to the manufacturer's instructions. Briefly, cells were harvested and washed in ice-cold phosphate-buffered saline (PBS), resuspended in 300 μL of binding buffer and incubated with 5 μL of Annexin V-FITC solution for 30 min at 4°C in the dark. This was followed by incubation with 5 μL of propidium iodide (PI) for 5 min. The samples were immediately analyzed by bivariate flow cytometry on the BD FACSCanto II instrument, equipped with Cell Quest software (BD). Approximately 1-5 × 10^5^ cells were analyzed in each sample (23).

### Colony Formation Assay

A colony formation (clonogenic) assay was used in order to determine cell survival. Briefly, remaining U87 MG cells and U251 cells after indicated treatments were trypsinized and plated into 6-well plates. The density of per well is 1,500 cells. Colonies were allowed to grow (~9–10 days). Fixed cells were then stained with a 0.5% (v/v) crystal violet (Sigma-Aldrich) solution. Only colonies consisting of ≥50 cells were counted as previously described (24).

### Transwell Migration Assay

DAPI labeled U87 MG cells and U251 cells were plated in the upper compartment of 0.8 μm Transwell chambers. After 6–8 h migration to the underside of the top chamber, a fluorescence microscope was applied to evaluate the migration of the fluorescently-labeled U87 MG cells and U251 cells. Each experiment was performed in triplicate.

### Microarray

Cells were immediately lysed in 500 μl TRIzol reagent (ThermoFisher Scientific) and stored at −80°C before purification using a standard phenol-chloroform extraction protocol with the RNAqueous Micro Kit (ThermoFisher Scientific). The transcriptome was analyzed using an Affymetrix human microarray (ThermoFisher Scientific) and normalized based on quantiles.

### Quantitative Reverse-Transcription Polymerase Chain Reaction (qRT-PCR)

Total RNA from clonal cells was isolated using RNeasy spin columns (Qiagen), per the manufacturer's protocol. For reverse-transcription reactions, first-strand cDNA was synthesized using Superscript reverse transcriptase (Invitrogen), per the manufacturer's protocol. TaqMan probes (Applied Biosystems) were used to estimate the level of gene expression of miR-33a-5p, PTEN, BRCA1, NBS1, RAD50, and MRE11*. GAPDH* and *U6* were used as housekeeping genes (25). The primer sets (Invitrogen) used are listed in Table 1.

**Table 1:** Primer sequences.

**Genes**	**Sequences**
miR-33a-5p	F: 5′ - GATCCTCAGTGCATTGTAGTTGC−3′
	R: 5′ - CTCTGTCTCTCGTCTTGTTGGTAT-3′
U6	F: 5′- GCTTCGGCAGCACATATACTAAAAT−3′
	R: 5′- CGCTTCACGAATTTGCGTGTCA-3′
PTEN	F: 5′ - GCAGAAAGACTTGAAGGCGTA−3′
	R: 5′ - AGCTGTGGTGGGTTATGGTC−3′
BRCA1	F: 5′ - GGCTATCCTCTCAGAGTGACATTT−3′
	R: 5′ - GCTTTATCAGGTTATGTTGCATGGT−3′
NBS1	F: 5′ - TTGGTTGCATGCTCTTCTTG−3′
	R: 5′ - GGCTGCTTCTTGGACTCAAC−3′
RAD50	F: 5 ′-CTTGGATATGCGAGGACGA−3′
	R: 5′ - CCAGAAGCTGGAAGTTACGC−3′
MRE11	F: 5 ′- GCCTTCCCGAAATGTCACTA−3′
	R: 5′ - TTCAAAATCAACCCCTTTCG−3′
GAPDH	F: 5′- AGGAGCGAGACCCCACTAAC−3′
	R: 5′- GATGACCCTTTTGGCTCCA-3′
siRNA-PTEN	5′-AAAGAGATCGTTAGCAGAA−3′
siRNA-NT	5′-ACACGTCCGAACATACTAC−3′
miR-33a-5p mimic	GUGCAUUGUAGUUGCAUUGCA
miR-NC mimic	TTCTCCGAACGTGTCACGT

### Luciferase Reporter Assay

The 3′-UTR of PTEN was synthesized, annealed, and inserted into the SacI and HindIII sites of the pMIR-reporter luciferase vector (Ambion), downstream of the luciferase stop codon. To induce mutagenesis, the sequences complementary to the binding sites of miR-33a-5p in the 3′-UTR of PTEN (gguuuUGCUACUCUAAUGCAu) were replaced by gguuuUGCUACUCUUUACGUu. The constructs were validated by sequencing. U87 MG cells and U251 cells were seeded into a 24-well plate to perform the luciferase assay. After overnight culture, cells were co-transfected with the wild-type or mutated plasmid, and equal amounts of the miR-33a-5p mimic or the miR- negative control mimic (miR-NC mimic). Luciferase assays were performed using the Dual Luciferase Reporter Assay System (Promega) 24 h after transfection.

### The miR-33a-5p Overexpression

Before transfection with the miR-33a-5p mimic and negative control (NC) mimic, U87 MG cells and U251 cells were seeded into 6-well plates at a density of 1 × 10^5^ cells per well, and incubated for 12 h. For the overexpression of miR-33a-5p, cells were transfected with the miR-33a-5p mimic or the NC mimic (Invitrogen, Carlsbad, CA, USA) using the X-treme transfection reagent (Roche Applied Science, Penzberg, Germany), according to the manufacturer's protocol. Forty-eight hours after transfection, cells were harvested for further analysis. The transfection efficiency was analyzed by qRT-PCR.

### Small Interfering (si) RNA Transfection

The siRNAs were applied to knock down PTEN expression in U87 MG cells and U251 cells. A non-targeting siRNA was used as a negative control (Invitrogen). The target sequences were as follows: PTEN: 5′-AAAGAGATCGTTAGCAGAA-3′; and Control: 5′-ACACGTCCGAACATACTAC-3′. Transfection efficiency was detected by qRT-PCR and western blotting.

### Immunofluorescence

Briefly, cells were fixed by 3% paraformaldehyde and then permeabilized with 0.5% Triton X-100. Cells were then blocked by 5% goat serum, followed by incubation with primary antibodies. Cells were then incubated with fluorescent secondary antibodies and DAPI to stain the targeted proteins and the nucleus, respectively (8). The cells were then analyzed by fluorescence microscopy.

### Cell Cycle Assay

Further, 70% cold anhydrous ethanol was applied to fix the cells. Then, the cells were treated with propidium iodide (PI) (Sigma, St. Louise, MO, USA) and RNase A. A flow cytometer equipped with Cell Quest software was used to detect the cell cycle distribution.

### Statistical Analysis

Data were expressed as the mean ± standard deviation (SD). Differences among groups were tested by one-way analysis of variance, and comparisons between two groups were evaluated by the Student's *t*-test, using the SPSS package v19.0 (SPSS Inc., Chicago, IL, USA). A *P*-value of < 0.05 was considered statistically significant.

## Results

### The Immune Checkpoint Inhibitor Sensitizes Gliomas to Radiotherapy

**Figure 1:**
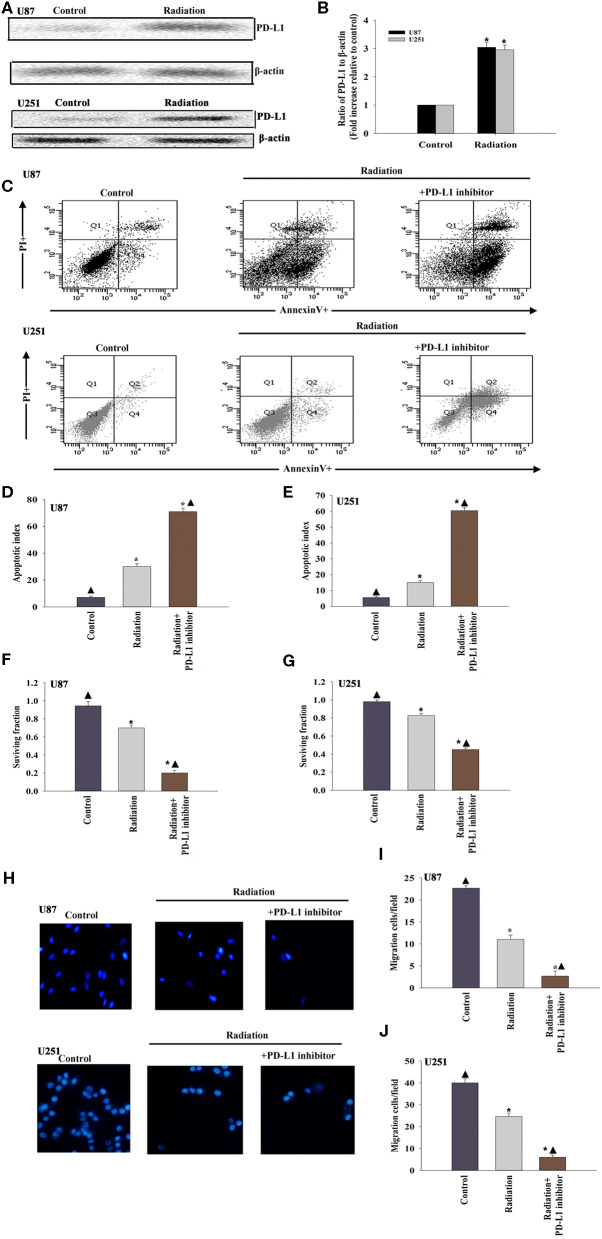
The immune checkpoint inhibitor sensitizes GBM to radiotherapy. **(A,B)** Representative images of western blots of PD-L1 and β-actin in radiated or untreated U87 MG cells and U251 cells. Fold changes were normalized to β-actin. Each column represents the mean ± SD from three independent experiments; *P < 0.05, vs. Control. U87 MG cells and U251 cells were subjected to radiation, with or without PD-L1-inhibitor treatment. Untreated U87 MG cells and U251 cells were used as the control separately. **(C)** Representative distributions of PI and Annexin V staining from FACScan flow cytometric analyses of apoptotic cells. **(D,E)** Percentage of apoptotic cells in above conditions. **(F,G)** Colony formation was presented as a bar graph in the U87 MG cells and U251 cells. **(H)** Fluorescence microscope images of the migrated U87 MG cells and U251 cells. **(I,J)** Data are presented as the number of migrated cells. Each column represents the mean ± SD from three independent experiments; *P < 0.05, vs. Control; ^▴^*P* < 0.05, vs. Radiation.

To determine whether the PD-L1 inhibitor would radiosensitize U87 MG cells and U251 cells, we first examined the expression of PD-L1 in the U87 MG cells and U251 cells under radiotherapy conditions. The results suggested that radiation induced PD-L1 expression (Figures 1A,B). We then administered the PD-L1 inhibitor two hours before radiation treatment and found that U87 MG cells and U251 cells were sensitized, as indicated by increased apoptosis (Figures 1C–E) and decreased cellular survival (Figures 1F,G). Cellular migration of the U87 MG cells and U251 cells was also inhibited by PD-L1 inhibitor (Figures 1H–J).

### Effect of the PD-L1 Inhibitor on miRNA Expression of Glioma Cells

**Figure 2:**
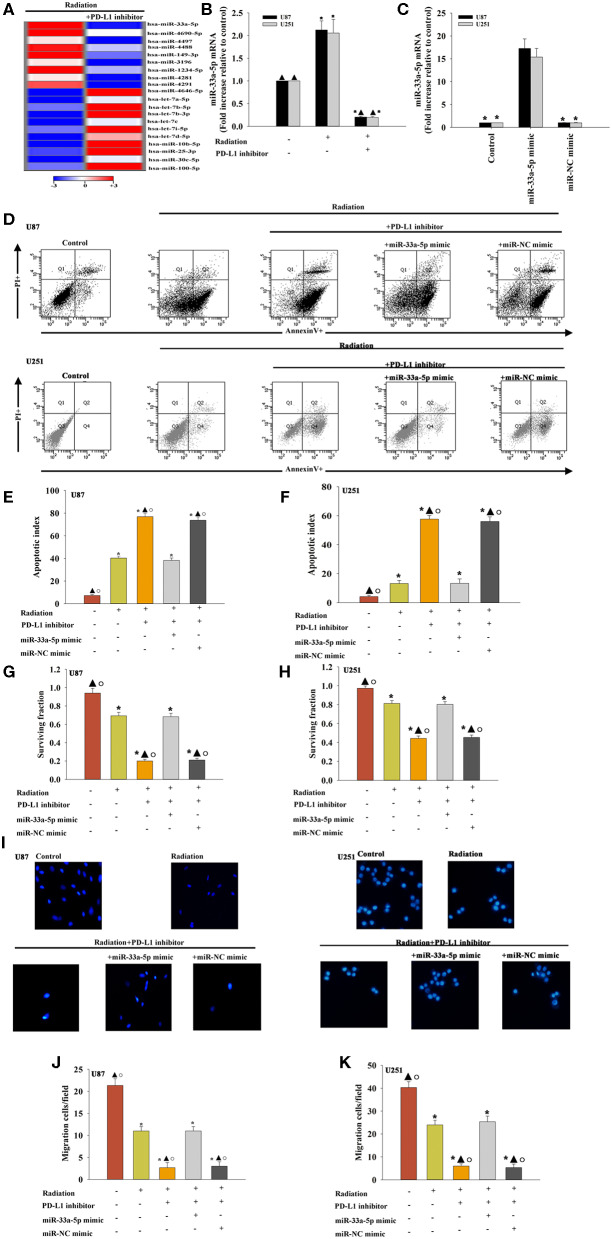
Effect of the PD-L1 inhibitor on miRNA expression in glioma cells. **(A)** Heat map of miRNAs differentially regulated by the PD-L1 inhibitor in radiated U87 MG cells. “Red” indicates up-regulation, and “blue” indicates down-regulation. **(B)** RT-qPCR validation of the differentially regulated miRNAs in U87 MG cells and U251 cells treated with radiation, with or without PD-L1 inhibitor pre-treatment. Untreated U87 MG cells and U251 cells were used as the control separately. *P < 0.05, vs. Control; ^▴^*P* < 0.05, vs. Radiation. U87 MG cells and U251 cells were transfected with a mimic control or the miR-33a-5p mimic, treated with the PD-L1 inhibitor, and subjected to radiation. In parallel, U87 MG cells and U251 cells, treated or untreated with the PD-L1 inhibitor were radiated. Un-treated U87 MG cells and U251 cells were used as the control separately. **(C)** The transfection efficiency was analyzed by qRT-PCR; *P < 0.05, vs. the miR-33a-5p mimic. **(D)** Representative distributions of PI and Annexin V staining from FACScan flow cytometric analyses of apoptotic cells. **(E,F)** Apoptotic cells in the above conditions. **(G,H)** Colony formation was presented as a bar graph in the U87 MG cells and U251 cells. **(I)** Fluorescence microscope images of the migrated U87 MG cells and U251 cells. **(J,K)** Data are presented as the number of migrated cells. Each column represents the mean ± SD from three independent experiments; *P < 0.05, vs. Control; ^▴^*P* < 0.05, vs. Radiation; °*P* < 0.05, vs. Radiation + PD-L1 inhibitor + miR-33a-5p mimic.

To examine the effect of miRNAs in the PD-L1 inhibitor-induced sensitization to radiotherapy, miRNA microarray probes were used. We found that the expression of specific miRNAs in–PD-L1 inhibitor treated before radiated U87 MG cells was significantly altered when compared with that in only radiated cells. Among them, miR-33a-5p was significantly downregulated in the PD-L1 inhibitor group, and therefore, we selected the down-regulated miR-33a-5p and verified the expression level using real-time PCR. The results showed that radiation induced increasing expression of miR-33a-5p, compared to the untreated cells. While, PD-L1 inhibitor decreased the expression of the miR-33a-5p (Figures 2A,B).

To determine the role of miR-33a-5p in PD-L1 inhibitor-induced radio-sensitization, U87 MG cells and U251 cells were transfected with the miR-33a-5p mimic (Figure 2C), and the negative control before treatment with the PD-L1 inhibitor. Compared to the radiation only group, treatment with the PD-L1 inhibitor induced more cellular apoptosis, while the overexpression of miR-33a-5p significantly inhibited apoptosis (Figures 2D–F). PD-L1 inhibitor treatment reduced the U87 MG cells and U251 cells surviving fraction compared to the radiation-only group; however, the overexpression of miR-33a-5p promoted the survival of U87 MG cells and U251 cells (Figures 2G,H). Transfection with the miR-33a-5p mimic dramatically promoted cell migration as well (Figures 2I–K). No apparent changes in the control miR-mimic were detected.

### PTEN Is a Direct Target of miR-33a-5p

**Figure 3:**
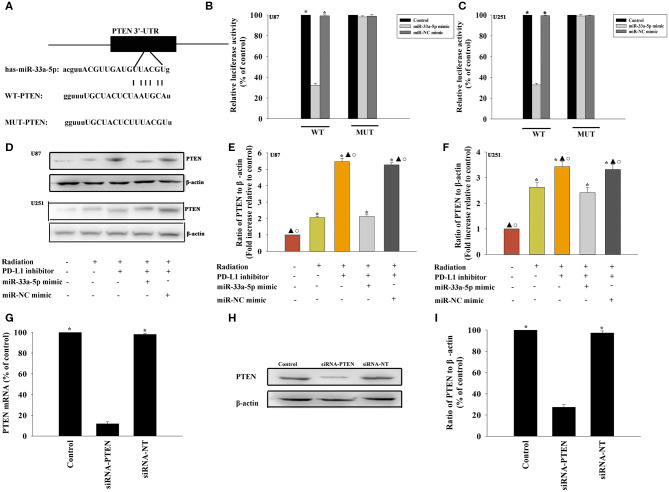
PTEN is a direct target of miR-33a-5p. **(A)** The predicted binding sites between miR-33a-5p and the PTEN 3′-UTR. **(B,C)** A dual luciferase assay was performed in U87 MG cells and U251 cells after co-transfection with PTEN 3′-UTR wild type (WT) or mutant (MUT) plasmids, miR-33a-5p mimics, and miR-NC mimic. *P < 0.05, vs. the miR-33a-5p mimic in the WT group; **(D–F)** western blot analysis of PTEN and β-actin protein levels in follow cells; U87 MG cells and U251 cells were transfected with a mimic control or the miR-33a-5p mimic, treated with the PD-L1 inhibitor, and subjected to radiation. In parallel, U87 MG cells and U251 cells, treated or untreated with the PD-L1 inhibitor were radiated. Untreated U87 MG cells and U251 cells were used as the control separately. *P < 0.05, vs. Control; ^▴^*P* < 0.05, vs. Radiation; °*P* < 0.05, vs. Radiation + PD-L1 inhibitor + miR-33a-5p mimic. **(G–I)** U87 MG cells were transfected with siRNA-PTEN, or with siRNA-NT as a control. siRNA-mediated transfection efficiency was determined by qRT-PCR **(G)** and western blotting **(H,I)**. Each column represents the mean ± SD from three independent experiments; *P < 0.05, vs. siRNA-PTEN.

To further explore the molecular action of miR-33a-5p in GBM tissues, we searched for potential miR-33a-5p targets using TargetScan. Bioinformatics databases were used to check the potential targets. PTEN is considered to be a putative target of miR-33a-5p (Figure 3A). Thus, GBM cells were co-transfected with a wild-type PTEN-luciferase reporter vector, together with the miR-33a-5p mimic or the miR-NC mimic, and tested luciferase activity. miR-33a-5p-transfected cells showed a remarkable reduction of luciferase activities from the PTEN reporter in U87 MG cells and U251 cells (Figures 3B,C). At meanwhile, compared with the mutated 3′-UTR, the luciferase activities of the cells transfected with the wild-type 3′-UTR showed significant reductions (Figures 3B,C). Next, western blot analyses were performed to evaluate PTEN protein expression. We found that the expression of PTEN was downregulated in the radiation group, while it was increased following treatment with the PD-L1 inhibitor. Overexpression of miR-33a-5p reversed the inducement of PD-L1 inhibitor (Figures 3D–F).

### The PD-L1 Inhibitor Confers Radiosensitization by Targeting PTEN

**Figure 4:**
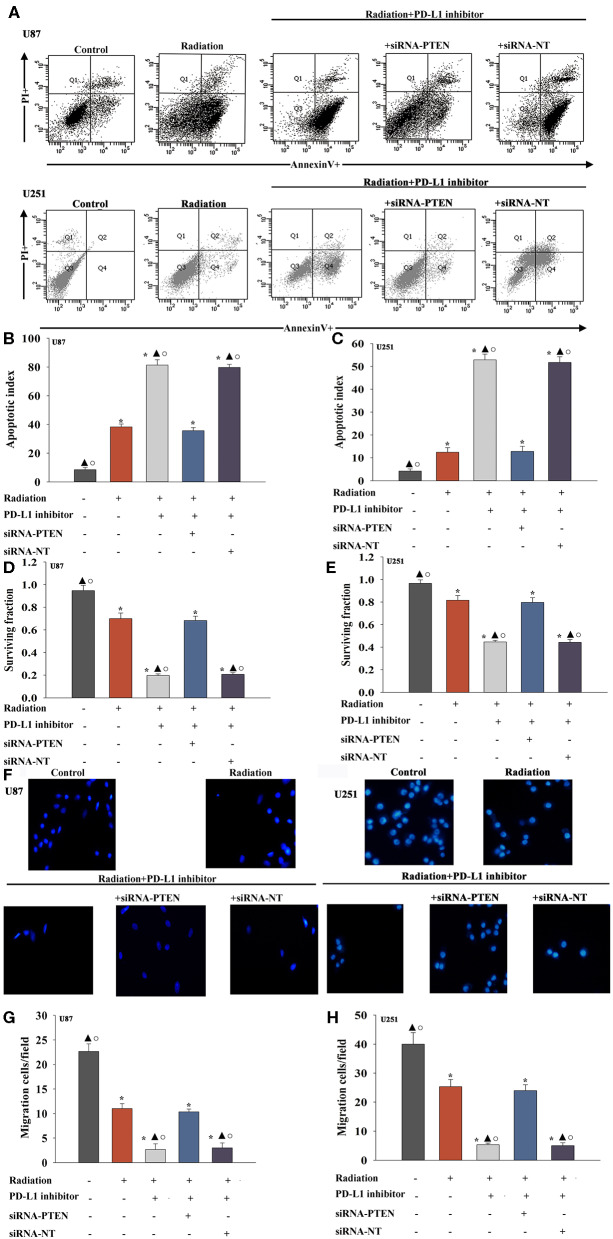
PD-L1 inhibitor confers radio-sensitization by targeting PTEN. U87 MG cells and U251 cells were transfected with siRNA against PTEN, or with siRNA-NT as a control, followed by treatment with the PD-L1 inhibitor and radiation. In parallel experiments, U87 MG and U251 cells were treated with radiation alone, or treated with the PD-L1 inhibitor in the presence of radiation. U87 MG and U251 cells under normal culture conditions were used as the control separately. **(A)** Representative distributions of PI and Annexin V staining from FACScan flow cytometric analyses of apoptotic cells. **(B,C)** Apoptotic cells in the above conditions. **(D,E)** Colony formation was presented as a bar graph in the U87 MG cells and U251 cells. **(F)** Fluorescence microscope images of the migrated U87 MG cells and U251 cells. **(G,H)** Data are presented as the number of migrated cells. Each column represents the mean ± SD from three independent experiments; *P < 0.05, vs. Control; ^▴^*P* < 0.05, vs. Radiation; °*P* < 0.05, vs. Radiation + PD-L1 inhibitor + siRNA-PTEN.

A previous study demonstrated that PTEN mediated the DNA damage response to radiosensitize high-grade gliomas (26). To assess whether the downregulation of PTEN reversed the PD-L1 inhibitor-mediated radio-sensitization, we used siRNA-PTEN to down regulate PTEN expression (Figures 3G–I). To test whether the PD-L1 inhibitor and PTEN have a role in cell apoptosis following radiation, we performed FACS analysis to determine the rates of cell apoptosis. The combined treatment of the PD-L1 inhibitor + radiation significantly increased cell apoptosis, compared to that observed following radiation only. As expected, the silencing of PTEN partially abolished the effect caused by PD-L1 inhibitor + radiation treatment (Figures 4A–C). Similarly, we also found that silencing PTEN partially reversed the inhibition of survival that was induced by the PD-L1 inhibitor + radiation treatment (Figures 4D,E). Our results also showed that PTEN silencing reversed the inhibition of U87 MG cells and U251 cells migration that was induced by the PD-L1 inhibitor (Figures 4F–H).

### The PD-L1 Inhibitor Induced DNA Damage and Reduced the DNA Damage Response to Take Radio-Sensitization Effect

**Figure 5:**
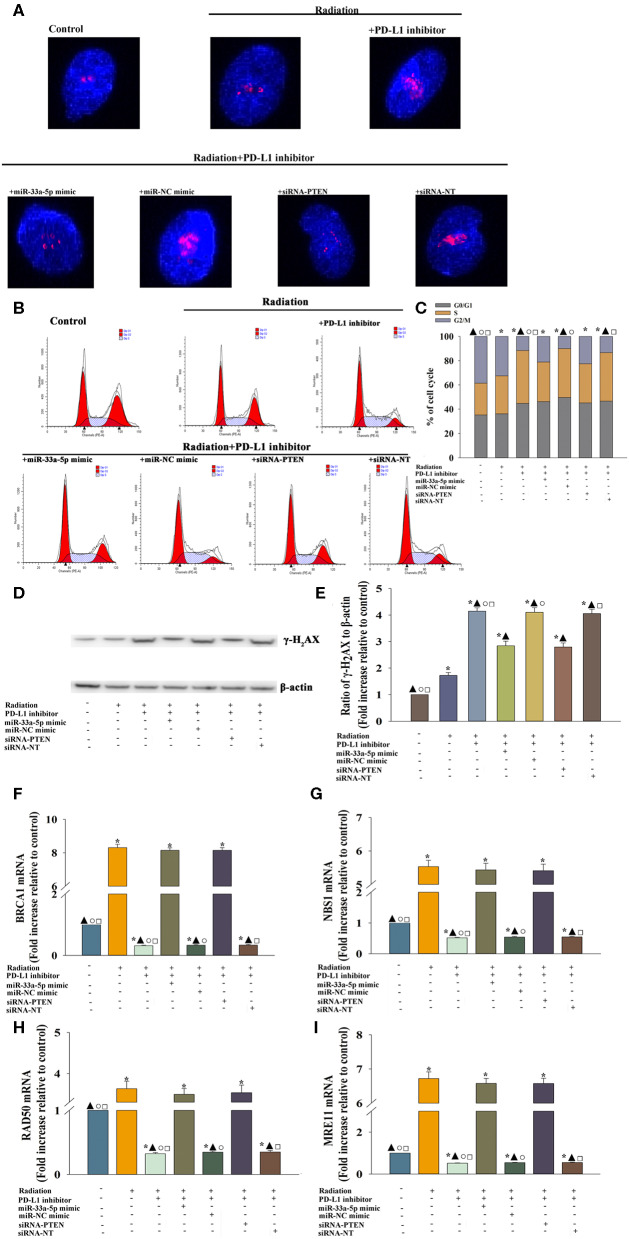
PD-L1 inhibitor-induced DNA damage and the reduced DNA damage response to take radiosensitization effect. U87 MG cells were transfected with siRNA against PTEN, or with siRNA-NT as a control, the miR-33a-5p mimic, or the miR-mimic control. Following transfection, cells were treated with the PD-L1 inhibitor in the presence of radiation. In parallel experiments, U87 MG were treated with radiation, or the PD-L1 inhibitor in the presence of radiation. U87 MG cells under normal culture conditions were used as the control. **(A)** The formation and resolution of γ-H_2_AX foci were assessed using immunofluorescence. **(B,C)** Cell cycle distribution was analyzed. **(D,E)** Western blot analysis of γ-H_2_AX and β-actin protein levels. **(F–I)** qRT-PCR analysis of BRCA1 **(F)**; NBS1 **(G)**; RAD50 **(H)**; and MRE11 **(I)**. *P < 0.05, vs. Control; ^▴^*P* < 0.05, vs. Radiation; °*P* < 0.05, vs. Radiation + PD-L1 inhibitor + miR-33a-5p mimic. ^□^*P* < 0.05, vs. Radiation + PD-L1 inhibitor + siRNA-PTEN.

We evaluated whether DNA repair could be impaired in cells treated with the PD-L1 inhibitor. Immunofluorescence was used to examine the kinetics of radiation-induced γ-H_2_AX foci following PD-L1 inhibitor treatment, which are indication of DNA double-strand breaks (Figure 5A). After PD-L1 inhibitor treatment, cells had higher basal levels of γ-H_2_AX, trapping in S-phase (Figures 5B,C), which was likely the result of the decreased repair of spontaneous DNA damage. PD-L1 inhibitor +radiation also increased the expression of γ-H_2_AX, compared with radiation alone (Figures 5D,E). However, either miR-33a-5p overexpression or silencing PTEN, could abolished the inducement of DNA damage by PD-L1 inhibitor (Figures 5A–E).

Then we searched to explore mechanistic insight into how the PD-L1 inhibitor affected the DDR. Thus, we assessed whether the important DDR pathway genes was affected by the PD-L1 inhibitor in U87 MG cells. We found that the expression of the DDR-related genes, BRCA1 (Figure 5F), NBS1 (Figure 5G), RAD50 (Figure 5H), and MRE11 (Figure 5I) were decreased following treatment with the PD-L1 inhibitor + radiation, compared to the levels of expression observed following radiation only. Additionally, there was a particularly reversed effect of DDR in the miR-33a-5p-overexpression or PTEN-silencing groups (Figures 5F–I).

## Discussion

GBM is the most common malignant primary central nervous system (CNS) tumor in adults, and is resistant to current therapies (27). Current evidence points toward the existence of a small fraction of tumor cells in the bulk tumor that also exhibit radio-resistant properties (28). Glioblastoma has been extensively studied as a paradigm for cancer-associated immunosuppression (29). Mount of immunosuppressive factors were existed on the glioma cell surface (30, 31). Importantly, PD-L1 was upregulated in the GBM microenvironment; thus, protecting GBM from T-cell killing (29). Our results revealed that GBM cells expressed PD-L1, and that radiation induced PD-L1 expression beyond that observed without radiation. H1A is a PD-L1 antibody that destabilizes PD-L1 by disrupting its binding with the PD-L1 stabilizer. Such destabilization results in greater PD-L1 degradation through the lysosome and sensitivity to radiotherapy is increased (8). Our results suggested that the inhibition of PD-L1, using a small molecular inhibitor, increased radio-sensitivity, which was indicated by increased apoptosis, decreased survive, and impaired migration in U87 MG cells and U251 cells.

In this study, our group is the first to report that the PD-L1 inhibitor repressed miR-33a-5p activity. We also demonstrated that the PD-L1 inhibitor was able to induce the expression of the PTEN by inhibiting miR-33a-5p, and further confer radiosensitization in GBM cells. To maintain the primary biological features of those cells, including stemness, self-renewal, and tumor initiation *in vivo*, the higher level of miR-33a-5p expression in GBM is required (32). Extensive research has been performed to demonstrate the important roles of miR-33a-5p in GBM initiation, progression, and recurrence associated with resistance to radiotherapy (14). Our results showed that radiation induced miR-33a-5p expression, which led to radiation resistance, while reversed by PD-L1 inhibitor. After delivering the PD-L1 inhibitor, we observed an upregulation of the miR-33a-5p target, PTEN. It has been reported that the loss of PTEN promotes gliomagenesis (33) and GBM radiation resistance (34). Similarly, PTEN silencing abolished the PD-L1 inhibitor-induced radiosensitization.

The inhibition of DNA repair was required to overcome radio-resistance (35). Thus, we found that the PD-L1 inhibitor was effective at radiosensitizing U87 MG cells by inhibiting the DDR. Other reports have confirmed the radiosensitizing potential associated with inhibiting the DDR at the pre-clinical level (36, 37). As report, the DDR recently has been confirmed to promote the radiation-induced upregulation of PD-L1 in tumor cells, increased exhaustion of CD8+ T cell induced by radiation, to achieve a greater pro-tumor response (38). Given the well-characterized GBM related immunosuppressive tumor microenvironment, treatment with PD-L1 inhibitors may present important weapons against this disease, such as targeting the DDR in GBM cells following radiation therapy (39, 40). The increasing number of γ-H2AX foci in the S-phase fraction commonly occurred in the DNA damage process (41). Similarly, our results showed that radiation induced the DDR in the U87 MG cells, while the PD-L1 inhibitor impaired the DDR, accompanied with increasing γ-H2AX foci and GBM cells trapping the S-phase; thus, leading to radiation sensitization.

In conclusion, the present study showed that the PD-L1 inhibitor induced radiation sensitization in U87 MG cells and U251 cells by directly targeting miR-33a-5p, activating the PTEN signaling pathway, and inhibiting the DDR process. These findings provide new insights into the understanding of the molecular mechanisms by which PD-L1 inhibitors mediate radiation sensitization in GBM.

## Data Availability Statement

The original contributions presented in the study are publicly available. This data can be found here: ArrayExpress (https://www.ebi.ac.uk/arrayexpress/) accession E-MTAB-9007.

## Author Contributions

WX, JZ, and YT made substantial contributions to the acquisition, analysis and interpretation of data. XWa, XWe, and XZ were the major contributors in writing the manuscript. MH and SL were involved in conception and design, revising it critically for important intellectual content. All authors read and approved the final manuscript.

## Conflict of Interest

The authors declare that the research was conducted in the absence of any commercial or financial relationships that could be construed as a potential conflict of interest.
